# The internal rotation and shift-test for the detection of superior lesions of the rotator cuff: reliability and clinical performance

**DOI:** 10.1016/j.jseint.2022.01.011

**Published:** 2022-02-18

**Authors:** Georg Fieseler, Kevin Laudner, Julia Sendler, Jakob Cornelius, Stephan Schulze, Wolfgang Lehmann, Souhail Hermassi, Karl-Stefan Delank, René Schwesig

**Affiliations:** a Clinic of Orthopedic and Trauma Surgery, Sports Medicine, Klinikum Hann, Münden, Germany; b Department of Health Sciences, University of Colorado Colorado Springs, Colorado Springs, CO; c Department of Orthopaedic and Trauma Surgery, Martin-Luther-University Halle-Wittenberg, Halle (Saale), Germany; d Clinic of Orthopedic, Trauma and Reconstructive Surgery, Georg August University Göttingen, Göttingen, Germany; e Physical Education Department, College of Education, Qatar University, Doha, Qatar

**Keywords:** Clinical test, Orthopedic exam, Shoulder, Rotator cuff, Validity, Reliability

## Abstract

**Background:**

Using reliable and valid clinical tests are essential for proper diagnosis and clinical outcomes among injuries involving the rotator cuff. The addition of a new clinical examination test could improve the clinical diagnosis and informative value of the sensitivity and specificity of pathology. This study of diagnostic accuracy evaluated the use of a new rotator cuff test, called the internal rotation and shift-test (IRO/shift-test), to determine its reliability and clinical performance (sensitivity, specificity, positive (PPV)/negative predictive value (NPV)). Clinical diagnostic outcomes were confirmed with radiological findings (MRI).

**Methods:**

100 patients from a specialized shoulder unit participated (64 male, 36 female, mean age: 55 ± 13.5 years). A single-blinded (no knowledge of prior clinical or technical diagnostics) study design was used with two experienced physicians performing the IRO/shift-test. For clinical performance, all clinical testing was compared with MRI.

**Results:**

The intra-rater (ICC = 0.73, 95% CI: 60-82) and inter-rater (ICC = 0.89, 95% CI: 81-94) coefficients for the IRO/shift-test showed good-to-excellent reliability. 75% of the patients showed a positive IRO/shift-test, while 65% had a radiologically diagnosed superior rotator cuff tear. 60% of these patients had both a positive IRO/shift-test and objective rotator cuff tear via MRI. The sensitivity of the IRO/shift-test to detect superior rotator cuff lesions based on MRI diagnosis was calculated at 92% (95% CI: 86-99%), while specificity was 67% (95% CI: 50-84%). Predictive values were also found to be high with 86% PPV (95% CI: 78-94%) and 80% NPV (95% CI: 64-96%).

**Conclusion:**

Our results demonstrate that the IRO/shift-test is a reliable and valid tool for assessing superior rotator cuff pathology. With good-to-excellent intrarater and inter-rater reliability and strong sensitivity and specificity this test should be considered a valuable addition to clinicians’ cadre of clinical evaluation tools.

Rotator cuff tears are one of the most common causes of pain and musculoskeletal complaints^25^^,^^33^ affecting individuals of all ages. Approximately 34% of asymptomatic individuals have a rotator cuff tear, while frequency continues to increase with age.^30^ Although some individuals can continue with normal and pain-free activities of daily living,^30^ the majority of these patients experience pain, loss of mobility, and loss of function resulting in medical bills ranging from $19,000 to $40,000.^21^

During clinical examinations, clinicians use functional, range of motion, and muscle tests to determine joint stability, available motion, and force production.^13^ Common special tests for identifying superior rotator cuff lesions include, but are not limited to, Jobe’s test, Hawkin’s sign, drop arm test, full can test, Neer’s sign, and the external rotation lag sign. Unfortunately, the diagnostic accuracy of these tests range drastically.^18^^,^^20^^,^^34^ As such, there is still confusion on the benefits of using these specific special tests. The effectiveness of these clinical tests are based on the specific scientific conditions under evaluation, statistically calculated evidence like sensitivity, specificity, positive predictive value (PPV), and negative predictive value (NPV) and must be in accordance with the anatomic structures tested.^4^^,^^16^ For these reasons, a clinical diagnosis following an examination is based on the summation of findings from multiple tests and not on an individual test.^14^^,^^18^ As such, a new clinical shoulder test that adequately addresses reliability and validity could be added to clinicians’ existing examination protocols to improve clinical diagnoses and patient outcomes.

The objective of this study was to determine the intra-reliability and inter-reliability, sensitivity, positive predictive value, and negative predictive value of the internal rotation and shift-test (IRO/shift-test). To determine the reliability of the test, multiple clinicians performed the test on the same patients on multiple occasions. For determining the effectiveness (sensitivity and specificity) of this test, clinical findings were correlated with radiological descriptions of the affected shoulder. We hypothesized that the IRO/shift-test would be a reliable and clinically valid tool for assessing superior rotator cuff tears.

## Methods

### Study protocol

A total of 100 patients from a specialized shoulder orthopedic unit were included in this study from a sample of convenience (64 male, 36 female, mean age: 55 ± 14 years, height: 1.75 ± 0.08 m, weight: 85 ± 17 kg, body mass index: 28 ± 5 kg/m^2^). All subjects were 18 years of age or older and were seen between 10/2018 and 11/2019. Patients who were status post posterior dislocation, arthrosis, fracture, arthritis, adhesive capsulitis, or restricted ROM were excluded. This study was approved by the Ethics Commission of the Martin-Luther University Halle-Wittenberg (reference number: 2018-05). All subjects provided informed consent prior to the examination and any data collection. This study complies with the Standards for Reporting of Diagnostic Accuracy (STARD).

We used a prospective, single-blinded study design where the investigators did not have any prior clinical or technical diagnostic information of each patient. Following the collection of each patient’s history, a standardized and independent clinical examination was performed separately by two experienced physicians. Neither physician was informed of the other physicians’ clinical findings. All clinical assessments were performed bilaterally and in the same order. For intra-rater reliability, one of the physicians repeated their clinical examination, with at least 1 week in between the two testing sessions. For inter-rater reliability, the findings of the two physicians performed on the same patients were compared. These procedures for evaluation of the inter-rater and intra-rater reliability have been previously defined.^10^^,^^11^^,^^15^ Magnetic resonance imaging (MRI) was then completed for all 100 patients (average time between clinical examination and MRI was approximately 5 weeks). MRI findings were analyzed by an experienced and external radiologist who was independent of the intention of the study and had no knowledge of clinical findings. All partial and full-thickness tears were considered positive for a rotator cuff lesion.

### IRO/shift-test

**Figure 1:**
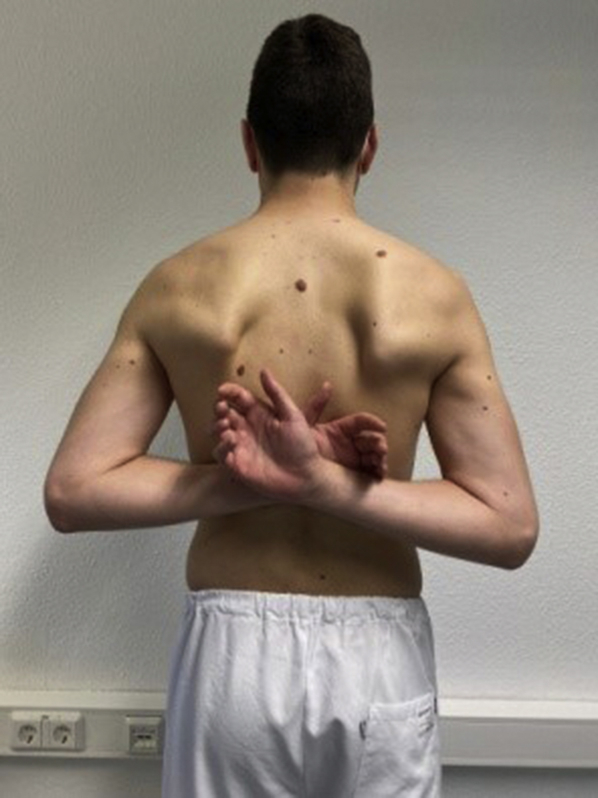
First step of IRO/shift-test, bilateral active shoulder internal rotation.

**Figure 2:**
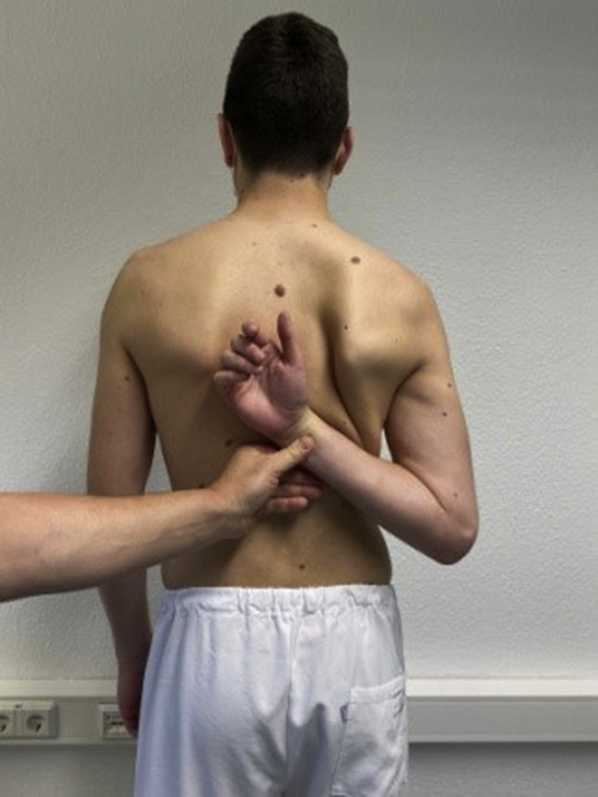
Second step of IRO/shift-test, clinician applied passive shoulder adduction and internal rotation.

Clinical examinations of the glenohumeral joint often include internal rotation mobility by assessing how far the patient can superiorly reach their involved hand along their spine. In this process, qualification and quantification are achieved by assessing the patient’s active and pain-free movement of the hand/arm cranially along the spine. More specifically, the individual vertebral body spinous process that can be reached by the upper edge of the patient’s finger is compared bilaterally to determine internal rotation capacity. Similarly, for the IRO/shift-test, we began with the patient actively moving his/her involved side of the dorsal hand along the spine in a cranial direction (Fig. 1). However, for the IRO/shift-test, at the end of this active motion, the clinician then passively provided overpressure, moving the arm into greater adduction and internal rotation (Fig. 2). This higher degree of passive adduction and internal rotation causes the humeral head to shift anteriorly, thus placing tension on the superior rotator cuff. The test was considered positive if this increased passive motion caused pain or discomfort at the anterior shoulder. If painful, the clinician then ruled out any involvement of the long head of the biceps, which may also be stressed during the motions of this test. If follow-up biceps testing, such as the O’Brien’s test, were negative, then a superior rotator cuff lesion was suspected.

### Statistical analyses

Intraclass and interclass correlation coefficients (ICC) were calculated to evaluate the intra-rater and inter-rater reliability.^28^ Interpretation of ICC values were based on several recommendations.^15^^,^^23^^,^^31^ ICC values were considered ‘excellent’ if greater than 0.75, ‘fair to good’ if between 0.40–0.75, and ‘poor reliability’ if lower than 0.40.

Clinical test performance characteristics (sensitivity, specificity, PPV, NPV) were calculated using MRI findings as the gold standard for comparison. 95% confidence intervals were calculated using a normal approximation for sensitivity and specificity and methodology described by Koopman^19^ for likelihood ratios. In this context, sensitivity was defined as the total number of superior rotator cuff tears found on MRI with those who also had a positive IRO/shift-test (i.e. low number of false negatives). In contrast, specificity was defined as the total number of patients who had no signs for a rotator cuff lesion (negative) using MRI and who also had a negative IRO/shift-test (i.e. low number of false positives). The positive predictive value (PPV) was defined as the number of subjects who had a rotator cuff tear found using the IRO/shift-test and confirmed radiologically (i.e. true positive). The negative predictive value (NPV) was the probability that a patient who had a negative clinical test would also have negative MRI (i.e. true negative). Predictive values are related to both sensitivity and specificity. The greater the sensitivity of a clinical test, then the greater the likelihood of finding true positives (PPV). Whereas higher specificity leads to greater consistency of finding true negative results (NPV).^23^

All statistical analyses were performed using SPSS version 28.0 for Windows (IBM, Armonk, NY, USA).

## Results

**Figure 3:**
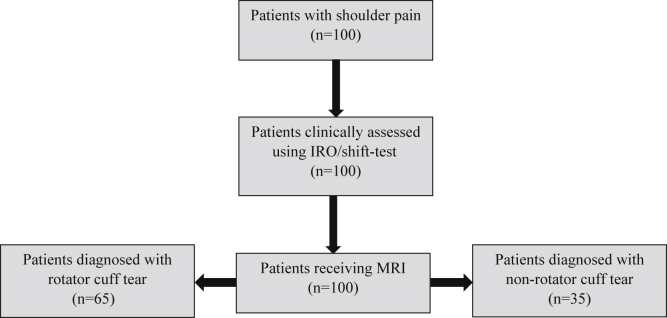
Participant flow diagram.

No participants were lost during the different testing protocols (Fig. 3), and no adverse events were reported.

### Reliability

The intra-rater (ICC = 0.89, 95% CI: 0.81-0.94) and inter-rater (ICC = 0.73, 95% CI: 0.60-0.82) coefficients for the IRO/shift-test showed good-to-excellent reliability (Table I).

**Table I:** Intra-rater and inter-rater reliability using the IRO/shift-test (n = 100).

Reliability	Descriptive statistics (mean ± SD)		ICC (95% CI)
	Observer 1	Observer 2	
Inter-rater reliability	0.75 ± 0.44	0.80 ± 0.40	0.73 (0.60-0.82)
	Session 1	Session 2	
Intrarater reliability	0.77 ± 0.43	0.83 ± 0.38	0.89 (0.81-0.94)

*SD*, standard deviation; *ICC*, intraclass/interclass correlation coefficient; *CI*, confidence interval.

### Comparison to MRI findings

Comparison of the IRO/shift-test with radiologically diagnosed superior rotator cuff lesions can be viewed in Table II. 75% of the patients showed a positive IRO/shift-test, while 65% had rotator cuff pathology found on MRI. 10 of these patients were diagnosed with a partial rotator cuff tear, while the remaining 55 had a full-thickness tear. Among the 35 patients who did not have a rotator cuff tear, there were a variety of pathologies found, including biceps tendon pathology, osteophytosis, bursitis, calcific tendinitis, AC joint arthrosis, and SLAP lesions. 60% of these patients had both a positive IRO/shift-test and objective rotator cuff tear via MRI; while only 15% had a positive IRO/shift-test and a negative finding on MRI. 5 patients presented with a positive IRO/shift-test and a subsequent positive follow-up O’Brien’s test resulting in an overall negative clinical diagnosis. The sensitivity of the IRO/shift-test to detect superior rotator cuff lesions based on MRI diagnosis was calculated at 92% (95% CI: 86-99%), specificity was 67% (95% CI: 50-84%). PPV and NPV were determined at nearly the same high level with 86% (PPV 95% CI: 78-94%) and 80% (NPV 95% CI: 64-96%).

**Table II:** Clinical performance of the IRO/shift-test compared to MRI (negative = 35, positive = 65).

Test	Sensitivity (95% CI)	Specifity (95% CI)	PPV (95% CI)	NPV (95% CI)
IRO/shift-test	92% (86-99)	67% (50-84)	86% (78-94)	80% (64-96)

*PPV*, positive predictive value; *NPV*, negative predictive value; *CI*, confidence interval.

## Discussion

Using reliable and valid special tests during a clinical examination is critical to the successful diagnosis and care of patients suffering from rotator cuff pathology. Unfortunately, no single clinical test has demonstrated enough sensitivity or specificity to be used alone. Therefore, it is imperative that clinicians use a summation of clinically proven techniques during their clinical examinations. The results of this study demonstrate that the IRO/shift-test is a reliable and valid technique in the evaluation of superior rotator cuff pathology and may be beneficial when combined with subsequent special tests.

We believe the specific benefit of the IRO/shift-test is the force applied to the anterosuperior rotator cuff structures while applying passive overpressure to an adducted and internally rotated shoulder. In this position, the humerus begins to translate anteriorly, placing increased tension on any potentially damaged anterolateral soft tissues structures, such as the supraspinatus tendon. As elevation (shoulder adduction and internal rotation) increases, and so does the force applied to these structures, increased pain and discomfort suggest a positive test for superior rotator cuff pathology. From an anatomical viewpoint, the pathology of the long head of the biceps tendon could also potentially interfere with this translation and provide a false-positive result. As such, involvement of the biceps must be excluded with subsequent specific clinical tests, such as the O’Brien’s, Speed’s and biceps resisted flexion tests.^1^ However, some biceps pathologies, such as bicipital peritendinous effusion, have been associated with certain rotator cuff pathologies (e.g., supraspinatus tears), so this should be considered in the final evaluation.^17^

With advances in medical technology, some clinicians have speculated that clinicians have grown accustomed to relying more on advanced imaging and less on their own clinical examination.^9^ Proponents of focusing on these advanced imaging devices argue that the increased availability and percision of this type of testing, time constraints, and a decreased confidence in clinicians’ exam skills outweigh the amigous findings that may come from a physical exam.^7^ This conflicts with the consensus statement completed by the Upper Extremity Committee of the International Society of Arthroscopy, Knee Surgery and Orthopaedic Sports Medicine, which states that the first key to understanding shoulder pathology is a proper physical examination.^2^ Similarly, others argue that although laboratory testing is important, the omission of clinical findings can lead to inaccurate diagnosis and increased patient expenses.^5^^,^^7^^,^^24^ Fortunately, many studies investigating the diagnositc accuracy of a special test provide explanations of test values by reporting sensitivity, specificity, as well as positive and negative predictive values.^7^ This is why we felt it was important to include these variables in our study and support the use of the IRO/shift-test during rotator cuff evaluations.

Similar to all clinical tests, we recommend that the IRO/shift-test not be used in isolation. Numerous studies have reported on the improved diagnostic accuracy when combining multiple clinical tests during examination.^6^^,^^8^^,^^12^^,^^20^^,^^29^^,^^32^ One study, conducted by Summerville et al.^32^ tested 139 patients and found that no single test was highly sensitive for diagnosing rotator cuff pathology. However, these authors did note that using a combination of clinical tests improved diagnostic accuracy and is highly recommended for clinical examinations. Similarly, manual muscle testing alone may not fully detect tears. Nagatomi et al.^22^ administered manual muscle tests to a group of patients already diagnosed with either a rotator cuff tear, superior labrum anterior-to-posterior lesion, or a Bankart lesion and found that patients with less than 60% of the uninvolved side strength were diagnosed as positive for a muscle tear, while none with equal strength to the contralateral side were positive. These authors also reported that there was a mixture of positive and negative findings among patients with 60–99% of the contralateral muscle strength. These sporadic findings led the authors to suggest that a manual muscle test alone is very limited in the information it can provide and cannot be used to fully detect the muscle tears. Although the results of our study found that the IRO/shift-test was both reliable and valid for assessing superior rotator cuff pathology, we strongly recommend that this test be used in addition to an array of clinical tests preferred by the clinician.

There are a few limitations of our study worth noting. First, only two orthopedic physicians participated in the reliability and validity of our study. Results may vary based on the experience and level of comfort performing the IRO/shift-test and findings should be considered conservatively until the clinician is comfortable with the test procedures. Next, clinicians should be considerate of patients with restricted ROM, like those with adhesive capsulitis, as they may have pain or the inability to be passively moved into internal rotation and adduction. This also poses the question of why we used an internal rotation and adduction passive motion to elicit stress on the superior rotator cuff and why not use other motions, such as glenohumeral extension? However, we believe that due to the oblique anatomical direction of the supraspinatus tendon, the combined motions of internal rotation and adduction created during the IRO/shift-test may cause more stress to the superior rotator cuff than during a more singular plane motion, like extension. More specifically, the supraspinatus tendon runs in a medial-dorsal to lateral-ventral direction creating a trapezoid shape at its insertion onto the greater tuberosity.^26^ However, future research is necessary to prove our hypothesis. Lastly, some clinicians have speculated that even comparing clinical test findings to advanced imaging, such as an MRI, can be inconclusive.^27^ One study reported that among a group of 123 patients, abnormal MRI findings were highly prevalent in both their symptomatic and asymptomatic shoulders.^3^

## Conclusion

The results of our study demonstrate that the IRO/shift-test is a reliable and valid technique for assessing the integrity of the superior rotator cuff. However, this test may not be administrable among patients with extreme limitations in ROM. As with any clinical examination, we recommend that this test be used in summation with several other preferred tests and advanced imaging for increased diagnostic accuracy.

## Disclaimers

Funding: No funding was disclosed by the authors.

Conflicts of Interest: The authors, their immediate families, and any research foundation with which they are affiliated have not received any financial payments or other benefits from any commercial entity related to the subject of this article.
